# Optical coherence tomography automated layer segmentation of macula after retinal detachment repair

**DOI:** 10.1371/journal.pone.0197058

**Published:** 2018-05-07

**Authors:** Kyu Jin Han, Young Hoon Lee

**Affiliations:** Department of Ophthalmology, College of Medicine, Konyang University, 685 Gasuwondong, Seo-gu, Daejeon, South Korea; Massachusetts Eye & Ear Infirmary, Harvard Medical School, UNITED STATES

## Abstract

**Purpose:**

To investigate the thickness of retinal layers and association with final visual acuity using spectral-domain optical coherence tomography (SD-OCT) in macular area of macula-off rhegmatogenous retinal detachment (RRD) patients after a successful macular re-attachment.

**Methods:**

In retrospective study, a total 24 eyes with macula-off RRD were enrolled. All patients underwent vitrectomy to repair RRD. Outer plexiform layer (OPL), outer nuclear layer (ONL), photoreceptor layer (PR), retinal pigment epithelium (RPE) thicknesses were measured by the Spectralis (Heidelberg Engineering, Heidelberg, Germany) SD-OCT with automated segmentation software. The relationship between the thicknesses of each retinal layer and postoperative logarithm of the minimum angle of resolution scale (LogMAR) visual acuity was analyzed.

**Results:**

OPL and RPE thicknesses were not significantly different between the retinal detachment eyes and fellow eyes (P = 0.839, 0.999, respectively). The ONL and photoreceptor thickness were significantly thinner in the retinal detachment eyes (P <0.001 and 0.001, respectively). In the univariate regression analysis, preoperative best corrected visual acuity (BCVA), ONL thickness and photoreceptor thickness showed association with the postoperative BCVA (P = 0.003, <0.001 and 0.024, respectively). In final multiple linear regression model, ONL thickness was the only variable significantly associated with postoperative BCVA (P = 0.044).

**Conclusions:**

Segmented ONL and photoreceptor thickness of retinal detachment eyes were significantly thinner than fellow eyes. Segmental analysis of the retinal layer in macular region may provide valuable information for evaluation RRD. And ONL thickness can be used as a potential biomarker to predict visual outcome after RRD repair.

## Introduction

Rhegmatogenous retinal detachment (RRD) is the most common type of retinal detachment. RRD is caused by the movement of liquefied vitreous gel into the subretinal space through a retinal break driven by vitreoretinal traction and sequential separation of sensory retina and retinal pigment epithelium (RPE) [1]. The annual incidence of RRD is known to be about 10.5 people per 100,000 population [2]. The goal of RRD repair is to close the retinal break, eliminate the vitreous traction and thus enable re-attachment of detached retina. Although various surgical techniques such as scleral buckling, cryopexy, laser photocoagulation and vitrectomy are performed, the postoperative functional success rate is only about 30–80% [3–5]. Even after successful retinal reattachment, many patients complain of incomplete visual acuity, abnormal color perception and metamorphopsia [6–8]. It is difficult to determine the presence of complete anatomic reattachment and change in the macula using only funduscopic examination.

Optical coherence tomography (OCT) can produce a good resolution of the tissue monolayer through a time delay of the echo reflected from eye tissue using infrared rays and has been widely used for general retinal diseases and for the exam after retinal detachment surgery [9]. Through OCT, microstructure changes of the retina after retinal detachment surgery and various forms of preoperative retinal detachment have been studied [10–13]. In this regard, in vivo thickness measurement of the retinal layer with spectral-domain optical coherence tomography (SD-OCT) can be used to detect the changes from several retinal diseases [14, 15]. The new segmentation software designed for the Spectralis SD-OCT (Heidelberg Engineering, Heidelberg, Germany) enables the independent quantification of all the retinal layers in the macula. This differentiation also may contribute to improving our knowledge of the change after retinal re-attachment.

The purpose of this study was to investigate the thickness of retinal layers and association with final visual acuity using SD-OCT in macular area of macular-off RRD patients after a successful macular re-attachment.

## Patients and methods

### Study design

We retrospectively reviewed the medical records of 24 patients who underwent vitrectomy for macula-off RRD between December 2013 and January 2015, and were followed up for at least 6 months after repair. The protocol for this retrospective study was approved by the Institutional Review Board of Konyang University Hospital (2017-07-016) and the procedures used conformed to the tenets of the Declaration of Helsinki. Written informed consent was obtained from all patients after they were provided with information on the procedures.

Eyes with macular disorder such as preoperative epiretinal membrane, macular hole and macular degeneration, proliferative vitreoretinopathy and traumatic retinal detachment were excluded. In order to exclude the effects of high myopia, the eyes of high myopia over an axial eye length 27 mm were excluded. Eyes with complications, such as persistent subretinal fluid, recurrent retinal detachment, poor OCT images (with a signal strength lower than 20) were excluded. The cases of vitrectomy combined with encircling were included.

Comprehensive ophthalmologic examinations were performed at preoperatively and at every visit postoperatively. The duration of macular detachment was based on the onset of visual decrease. Best corrected visual acuity (BCVA) was measured using the Snellen Eye charts and then were converted to the logarithm of the minimum angle of resolution scale (LogMAR). We also examined retina preoperatively and recorded the number of retinal tear. We measured the axial lengths to exclude bias caused by axial length difference from fellow eyes. Axial lengths were measured using IOL Master^®^ (Carl Zeiss Meditec, Dublin, CA, USA) after confirmation of retinal re-attachment.

### Retinal detachment repair

For vitrectomy, total vitrectomy was performed using the 25-gauge (Constellation^®^ vision system, Alcon^®^ Laboratories, Inc.) and then was followed by internal subretinal fluid drainage through fluid-air exchange and laser photocoagulation applied around retinal tears. Fluid-gas exchange (C_3_F_8_ or SF_6_) was performed on all eyes. If there was clinically significant lens opacity preoperatively, phacoemulsification and intraocular lens implantation were performed. All surgeries were performed by only one vitreoretinal surgeon (YHL).

### Spectral-domain optical coherence tomography measurements

**Fig 1:**
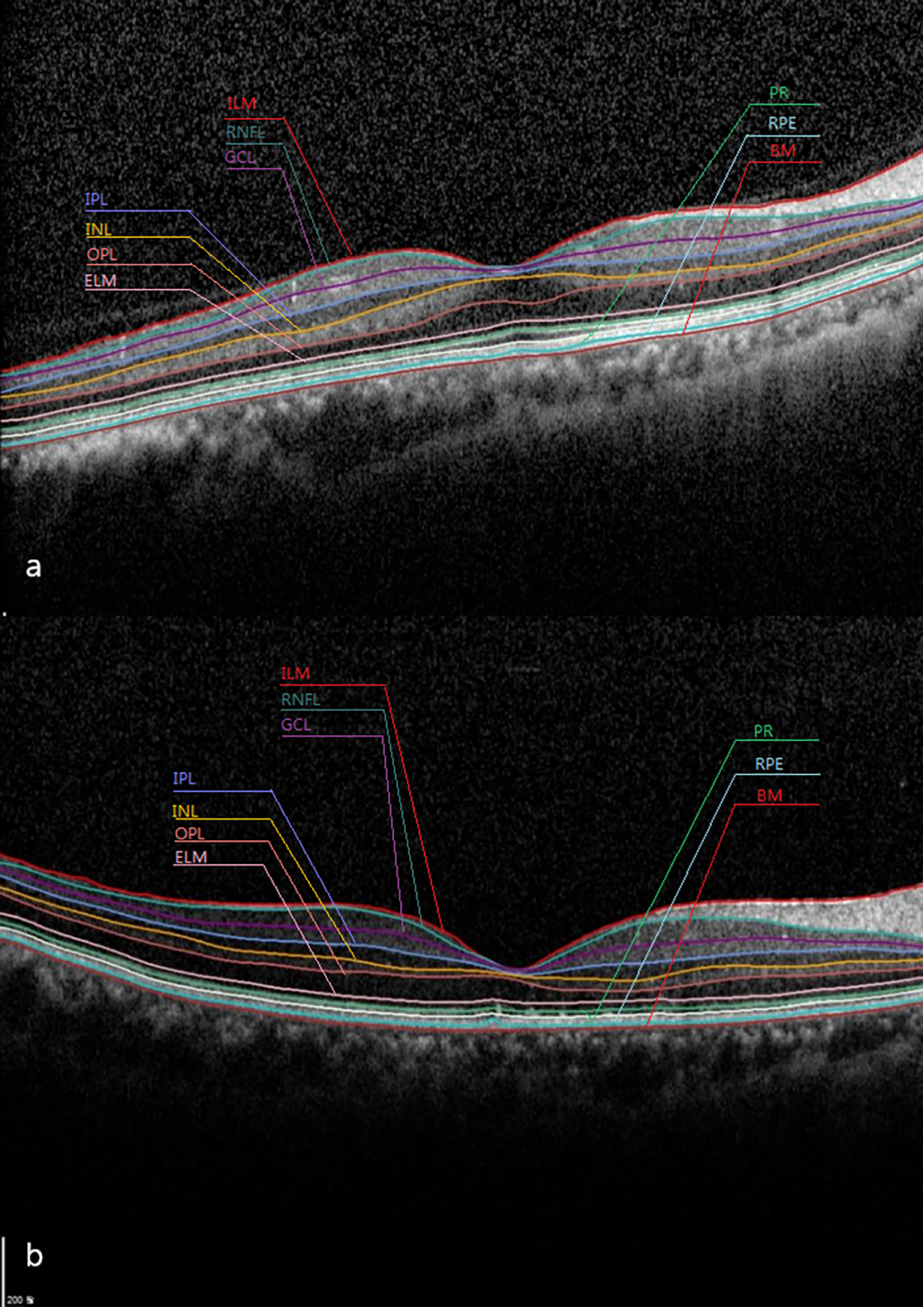
Automatically retinal layer segmentation by Spectralis software. Segmentation of the individual retinal layers can be seen in both healthy eyes (a) and retinal detachment eyes after surgical repair (b).

Spectralis SD-OCT incorporates real-time eye-tracking software (Heidelberg Eye Explorer^®^, version 1.8.6.0, Heidelberg Engineering, Heidelberg, Germany) has advantage of improving quality and segmentation accuracy to obtain retinal scans compromising 25 single horizontal axial scans in the macular area. The new Spectralis segmentation software was used to obtain individual retinal layer thickness measurements including: overall retinal thickness (RT), retinal nerve fibre layer (RNFL), ganglion cell layer (GCL), inner plexiform layer (IPL), inner nuclear layer (INL), outer plexiform layer (OPL), outer nuclear layer (ONL), RPE, inner retinal layer and photoreceptor layer (PR) (Fig 1).

**Fig 2:**
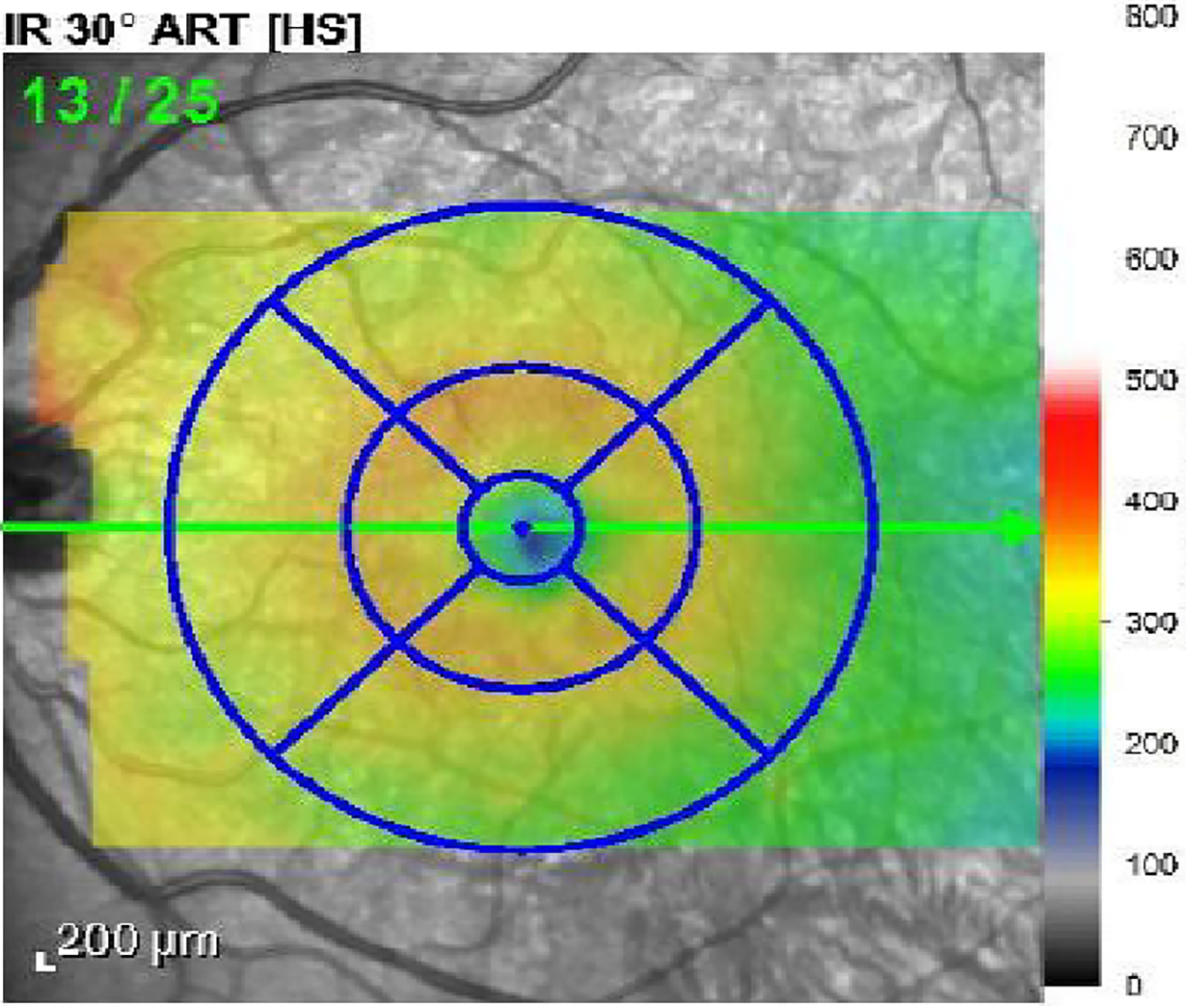
Retinal thickness analysis algorithm provide for each of 9 areas defined by an Early Treatment Diabetic Retinopathy Study (ETDRS) circle for every layer.

The foveal reflex center was identified as the frame containing a bright foveal reflex with the thickest outer segment. After automated segmentation of each retinal layer, the Spectralis mapping software generated automated measurements of thickness of each retinal layer on the central 1, 3, and 6 mm subfields as defined by the ETDRS (Fig 2). The thickness of each retinal layer within 1 mm ETDRS subfields was analyzed.

Fellow eye SD-OCT measurements were performed the day before surgery, and postoperative measurements were performed 6 months after surgical repair. If the measurements were available at 9 and 12 months after surgical repair, the data was used for analysis.

### Statistical methods

For statistical analysis, Wilcoxon signed rank test and regression analysis were performed using the SPSS for Windows software version 18.0 (SPSS, Inc., Chicago, IL, USA). When p-value was less than 0.05, it was determined to be statistically significant.

## Results

The demographics of subjects are summarized in Table 1.

**Table 1:** Demographic characteristics and clinical features of the patients.

Variables	Data
Subject eyes (n)	24
Mean age (y)	54.42 ± 9.79
Gender ratio, male:female	14:10
Preoperative BCVA (log MAR)	0.84 ± 0.29
Postoperative BCVA (log MAR)	0.40 ± 0.23
Macular detachment duration (day)	6.42 ± 4.49
Number of tear	2.00 ± 0.93
Preoperative lens status, Phakia: Pseudophakia	19:5
Tamponation gas, C_3_F_8_: SF_6_	18:6

Data are presented as the mean and standard deviation.

BCVA = best corrected visual acuity.

For the axial length, there was no significant difference between the fellow eyes and the retinal detachment eyes (P = 0.307). For the thickness of the OPL and RPE, there were no significant difference between the retinal detachment eyes and fellow eyes (P = 0.839, 0.999, respectively). For the thickness of the ONL, the retinal detachment eyes and fellow eyes were 80.17 ± 10.06 μm and 85.00 ± 9.45 μm, respectively. For the thickness of the PR, the retinal detachment eyes and fellow eyes were 84.25 ± 4.76 μm and 87.92 ± 4.27 μm, respectively. The thickness of the ONL and PR were significantly thinner in the retinal detachment eyes (P <0.001 and 0.001 respectively) (Table 2).

**Table 2:** Axial length and mean retinal layer thickness measurements.

	Retinal detachment eyes (Postoperative)	Fellow eyes	*P* value*
Axial length (mm)	24.93 ± 1.64	25.03 ± 1.33	0.307
OPL (μm)	28.92 ± 6.93	28.92 ± 7.20	0.839
ONL (μm)	80.17 ± 10.06	85.00 ± 9.45	<0.001
PR (μm)	84.25 ± 4.76	87.92 ± 4.27	0.001
RPE (μm)	15.71 ± 2.68	15.50 ± 1.72	0.999

Data are presented as the mean and standard deviation.

*Comparison between the groups by Wilcoxon signed rank test.

OPL = outer plexiform layer; ONL = outer nuclear layer; PR = photoreceptor; RPE = retinal pigment epithelium.

In the univariate regression analysis between postoperative BCVA and postoperative each retinal layer thickness with age, preoperative BCVA and retinal detachment duration, preoperative BCVA showed a significantly positive association (P = 0.003). Postoperative ONL thickness and photoreceptor thickness showed a significantly negative association (P < 0.001 and 0.024 respectively). In final multiple linear regression model, postoperative ONL thickness was the only significantly associated variable with postoperative BCVA (P = 0.044) (Table 3).

**Table 3:** Associations of postoperative BCVA with age, preoperative BCVA, macular detachment duration and postoperative each retinal layer thickness.

Variables	β ± SE	*P* value*	β ± SE	*P* value†
Age (y)	0.001 ± 0.005	0.969		
Preoperative BCVA (log MAR)	0.472 ± 0.141	0.003	0.256 ± 0.151	0.104
Macular detachment duration (day)	-0.004 ± 0.011	0.708		
Postoperative OPL (μm)	0.004 ± 0.007	0.607		
Postoperative ONL (μm)	-0.015 ± 0.004	<0.001	-0.012 ± 0.005	0.044
Postoperative PR (μm)	-0.023 ± 0.009	0.024	0.001 ± 0.010	0.968
Postoperative RPE (μm)	0.008 ± 0.018	0.667		

*Associations of postoperative BCVA with age, preoperative BCVA, macular detachment duration and postoperative each retinal layer thickness by univariate linear regression analyses.

†Associations of postoperative BCVA with preoperative BCVA, postoperative ONL thickness and postoperative photoreceptor thickness by multiple linear regression analyses.

SE = standard errors; BCVA = best corrected visual acuity; OPL = outer plexiform layer; ONL = outer nuclear layer; PR = photoreceptor; RPE = retinal pigment epithelium.

## Discussion

Retinal detachment causes the separation of both the sensory retina and RPE and leads to morphological changes in the retina and visual impairment. These changes cause macular edema and RPE disorder following anatomical re-attachment through surgery and leads to blurred vision, abnormal color perception and metamorphopsia due to the PR regeneration failure, PR misalignment and retinal pigment epithelial atrophic change even if changes in the macula are not able to be observed [16].

In this study, the average thickness of the ONL after surgery of retinal detachment was 80.17 ± 10.06 μm, which was significantly thinner than the average thickness of fellow eyes (85.00 ± 9.45 μm) (P < 0.001). According to an analysis of the thickness of each layer using a SD-OCT for the fovea 3 mm zone after surgery of macula-off retinal detachment, Marcel et al reported an increased the inner plexiform-outer plexiform layer thickness and a decreased the ONL thickness compared with fellow eyes [17]. According to an analysis of the ONL thickness after retinal detachment repair using a SD-OCT, Dooley et al reported that combined macular detachment revealed a thinner ONL thickness than normal eyes or macula-on retinal detachment eyes and that there was a significant correlation between the ONL thickness and postoperative visual acuity [18]. Also, Kim et al reported significant thinning of ONL in re-attached retina by measuring the thickness of retinal layers based on SD-OCT images [19]. The reduction in the ONL thickness may be the result of both ischemia caused by a blood supply disorder in the external layer after retinal detachment and apoptosis caused by cell loss in the ONL [20].

In this study, postoperative ONL thickness was the only significantly associated variable with postoperative BCVA (P = 0.044). This is consistent with previous studies that reported that the reduction in the thickness of both the ganglion cell-inner plexiform complex and ONL can be a predictor of the final visual acuity [17, 18].

For the PR thickness in this study, retinal detachment eyes had a significantly thinner thickness (84.25 ± 4.76 μm) than fellow eyes (87.92 ± 4.27 μm) (P = 0.001). Similar results have already been studied in many previouus reports. When retinal detachment occurs, damage to the PR and reduction in thickness lead to an incomplete visual recovery and delayed recovery of visual acuity. In retinal detachment, the distance from the choroid to the neuronal retina increases, then detached retina experiences a failure in blood supply form the choroid. The PR that needs a lot of oxygen and nutrients is vulnerable to the ischemia [21]. In animal model, retinal detachment leads to a reduction in PR outer segment absolute length and membrane assembly rates. The early morphologic changes are characterized by the clearance of subretinal debris, and the restoration of the morphologic relationship between the PR outer segments and the apical processes of the RPE [22]. Guerin et al reported degeneration of outer segments of both rod cells and cone cells occurred during retinal detachment in monkey model. Also they found that the thickness of outer segment of rod cells and the thickness of the outer segment of cone cells increased to 72% of normal thickness and 48% of normal thickness, respectively, through a gradual increase in the thicknesses 30 days after re-attachment [22]. With regard to changes in the PR layer after retinal detachment repair in humans, Terauchi et al reported that both the inner segment and outer segment had a significantly thinner layer in the first month after repair, compared with normal eyes [23]. Kim et al reported significant thinning of PR layer in re-attached retina in patients with relatively short durations of retinal detachment, the mean duration of retinal detachment was less than 1 week [19]. Even though a PR layer in the group with good visual acuity six months after surgery was reported to be thicker than PR layer in the group with poor visual acuity [23], there was no statistically significant correlation between postoperative LogMAR visual acuity and postoperative PR thickness in final multiple linear regression model (P = 0.968). This is thought to be due to differences in study methods, such as OCT equipment, analytical methods and the follow-up period.

It was assumed that the reduction in the RPE thickness after retinal detachment repair was caused by isolation of RPE in the separation process of the PR and lost RPE during drainage of subretinal fluid. There was no significant difference in the RPE thickness in both the retinal detachment eye and fellow eyes (P = 0.999).

An association between final visual outcome and patient age or retinal detachment duration have been found in other studies [24–26]. However, we could not found the relevance of retinal detachment duration and patient age to postoperative BCVA. This could be explained by the relatively short retinal detachment duration, 6.42 ± 4.49 days in this study.

An association between functional outcome and preoperative BCVA has been found in other studies and some authors have proposed preoperative BCVA is the most important variable related to the final visual result after retinal detachment surgery [26, 27]. Despite association between preoperative BCVA and postoperative BCVA was significant in the univariate regression analysis (P = 0.003), there was no significant association with postoperative BCVA in final multiple linear model (P = 0.104). Further well organized studies to control confounding factors like lens status are needed to confirm this result.

With regard to the limitations of this study, first, there were only a small number of subjects. Second, the inhomogeneity of the lens status and surgical methods. Regarding the cataract formation after vitrectomy, it may influence the final visual acuity. Third, we failed to reflect the changes in microstructure, because we just measured the thickness of individual retinal layers. And we could not confirm the sequential changes in the retina over time. Average postoperative observation point is too short to describe visual acuity and retinal status after the final surgery. Because morphological recovery and visual recovery can continue for six successive months after retinal re-attachment [28]. We conducted this study based on the assumption that the retina had stabilized and this would lead to less changes in forms and functions, when an average 7.37 ± 5.89 months had elapsed after re-attachment of the retina [22].

In conclusion, our findings indicate that with the new SD-OCT segmentation software, thickness of ONL and PR after retinal re-attachment were thinner than those of fellow eyes. ONL thickness was significantly associated with the postoperative BCVA after retinal detachment repair and can be used as a potential biomarker to predict visual outcome.

## Supporting information

S1 Table Demographics, ocular characteristics of participants and retinal layer thickness. (XLSX)
